# Sandwiching a Lumbar Hernia: A Case Report

**DOI:** 10.7759/cureus.71590

**Published:** 2024-10-16

**Authors:** Aiswerya Shankar, Karthikeyan Selvaraj, Prasanna Kumar, Sasikumar Pattabi

**Affiliations:** 1 General Surgery, Sree Balaji Medical College and Hospital, Chennai, IND; 2 Surgery, Sree Balaji Medical College and Hospital, Chennai, IND

**Keywords:** hernia repair, inferior lumbar triangle, open sandwich technique, primary lumbar hernia, ventral hernia presentation

## Abstract

A lumbar hernia is characterized by the protrusion of intra-abdominal contents through a defect in the muscles of the lumbar region. They can be classified as primary or secondary based on their etiology. We present a case of a 65-year-old male who had swelling in the right flank region for nearly 20 years and experienced sudden onset pain over the last 20 days. He was diagnosed with a primary lumbar hernia in the inferior triangle, which was confirmed by computed tomography. Open onlay mesh repair was performed using a recently developed technique known as the sandwich technique. In this method, the muscle and defect are sandwiched between two polypropylene meshes to improve tensile strength and provide support, preventing future recurrence. The patient was followed up at one week, one month, and six months post-procedure, with no recurrence or complications observed. This case report highlights a rare type of primary lumbar hernia and emphasizes the successful outcome of this novel repair technique.

## Introduction

A lumbar hernia is an unusual bulge where the abdominal contents herniate through the muscles of the posterolateral abdominal wall. It is a rare occurrence, accounting for only 1.5% of all hernias [1]. Lumbar hernias are categorized into two types, superior and inferior, based on their location. A superior lumbar hernia is situated above the transversus abdominis muscle, while the inferior lumbar hernia is found below it.

Lumbar hernias can occur due to congenital defects, surgical defects, or trauma. Symptoms include a visible protrusion in the flank, pain or discomfort, or vomiting. Surgical intervention is required to avoid complications such as obstruction, strangulation, or perforation. One in every four cases carries a risk of incarceration, and 8 in 10 cases are likely to involve strangulation [2]. Lumbar hernia typically presents as swelling or bulge in the lower back region, with pain when bending forward and discomfort when lying down. These symptoms may be exacerbated by physical activities such as lifting heavy objects, coughing, sneezing, or straining during bowel movements. Patients may also experience numbness or tingling in the back or legs. In severe cases, it can cause constipation or difficulty in passing urine. Computed tomography (CT) is the investigation of choice.

This article aims to present an uncommon case of a primary right lumbar hernia located in the inferior triangle and to introduce a new repair method known as the sandwich technique.

## Case presentation

A 65-year-old male, employed as a typist, presented to the surgery clinic with complaints of swelling in the right lumbar region for 20 years. The swelling had an insidious onset and gradually increased in size. It became more prominent when bending forward, getting out of bed, or lifting weights, and would reduce when lying down. He also reported a sudden onset of pain in the left lower back region over the past 20 days. The pain was dragging in nature, non-radiating, with no specific aggravating or relieving factors. He had no previous hospital visits for the same complaints nor a history of trauma or surgery to the area. He had been hypertensive for 10 years and had suffered from insomnia for seven years, for which he was on regular treatment and follow-up with his primary care physician. The patient did not have any other comorbidities. There was no history of tuberculosis. He reported occasional alcohol consumption for the past three years and was a non-smoker. There was no history of substance abuse. He consumed a mixed diet and had regular bowel and bladder habits. There was also no significant family history.

**Figure 1:**
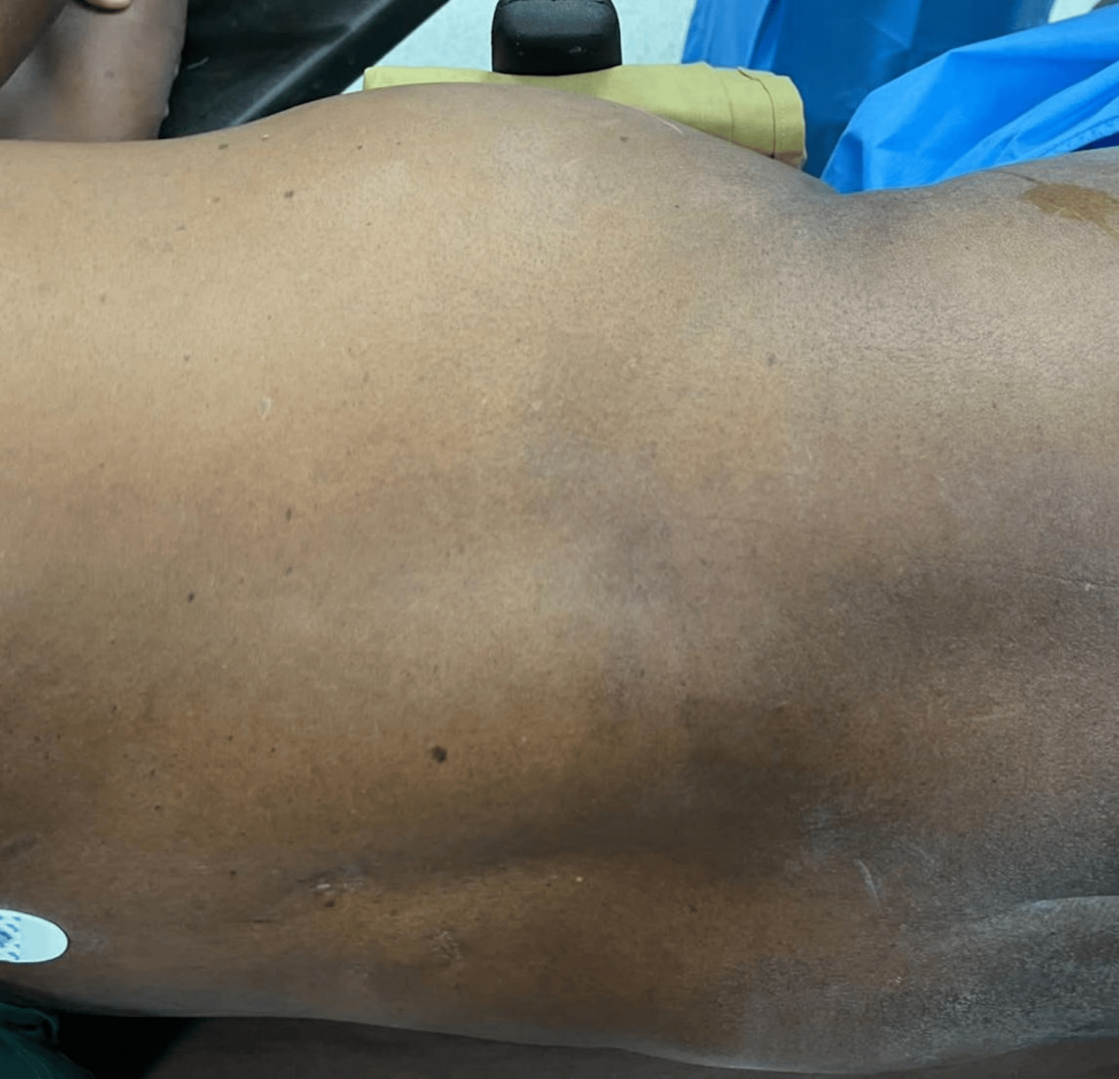
Lumbar hernia with the patient in the left lateral position

**Figure 2:**
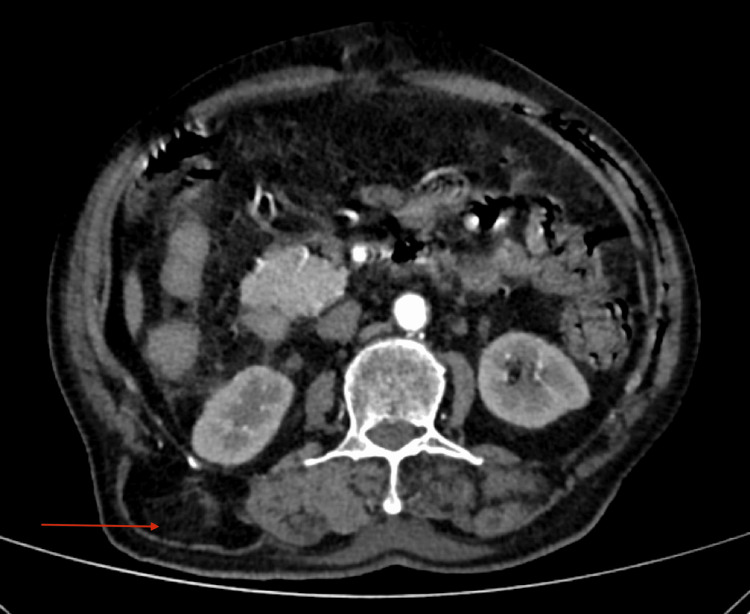
CT of the abdomen showing defect in the right posterior lumbar region

Upon general examination, the patient was well-built and well-nourished. After obtaining consent, the patient was examined in both supine and standing positions in a well-lit room. On physical examination, a single swelling measuring 8 × 6 cm was found in the right lumbar region. It extended 10 cm above the posterior superior iliac spine and 15 cm below the inferior angle of the scapula, with its lateral border positioned 2 cm from the midline. The swelling had a smooth surface, was ill-defined and soft in consistency, and reduced spontaneously when the patient lay in a supine position. There was no warmth or tenderness over the swelling. The plane of the swelling was found to be deep to the muscle, and when the muscle contracted, the swelling became less prominent. A cough impulse was present (Figure 1). There were no visible pulsations or peristalsis. When the patient raised his head or legs, no obvious swelling was visible. On percussion, a dull note was present over the swelling, and no bowel sounds were heard on auscultation. The patient was advised to undergo a CT of the abdomen, which showed a 3.4 cm defect in the right posterolumbar region with retroperitoneal fat as its content, confirming a right lumbar hernia (Figure 2).

**Figure 3:**
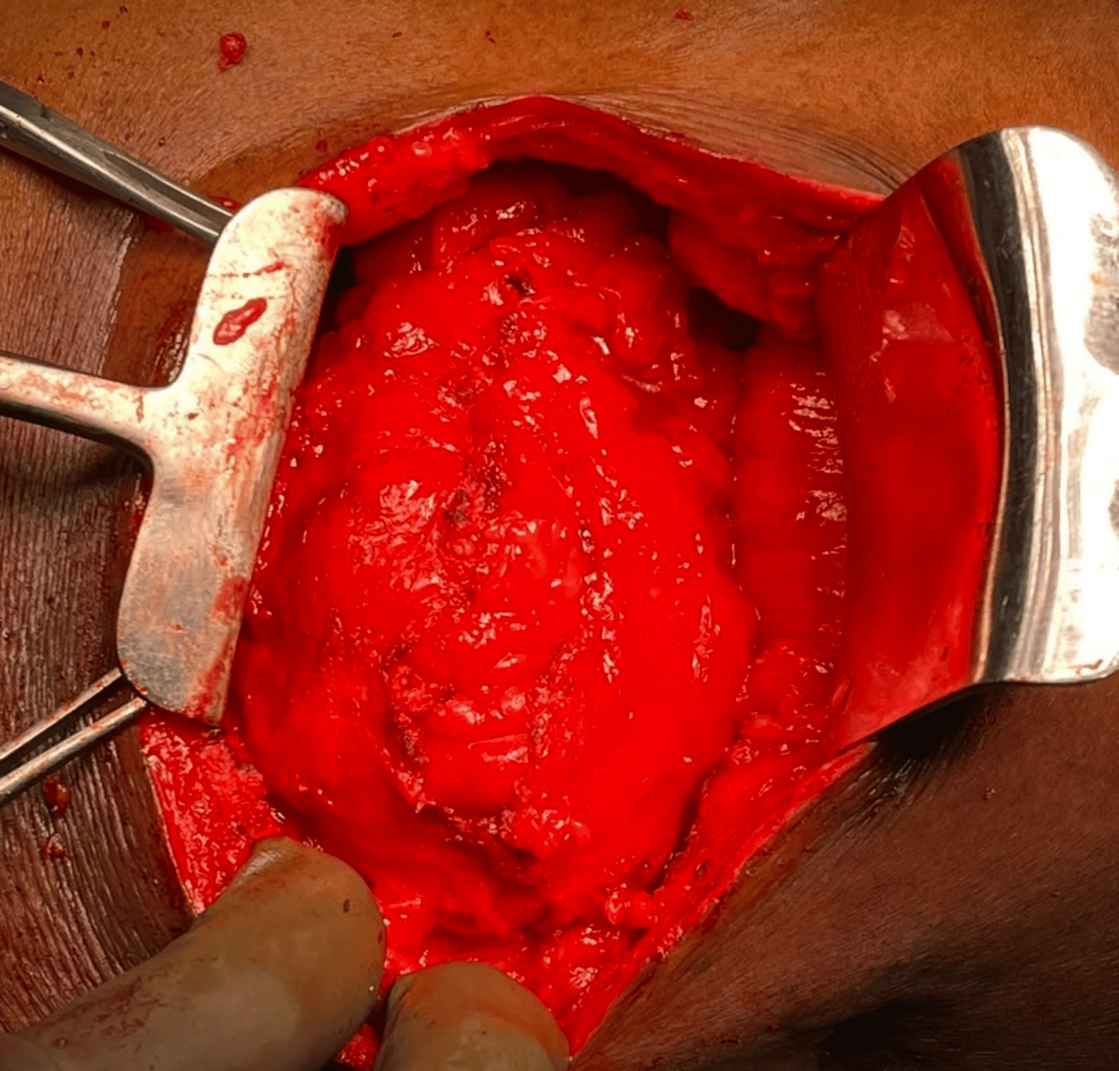
Contents (extraperitoneal fat and omentum) herniating through the defect intraoperatively in the inferior triangle

**Figure 4:**
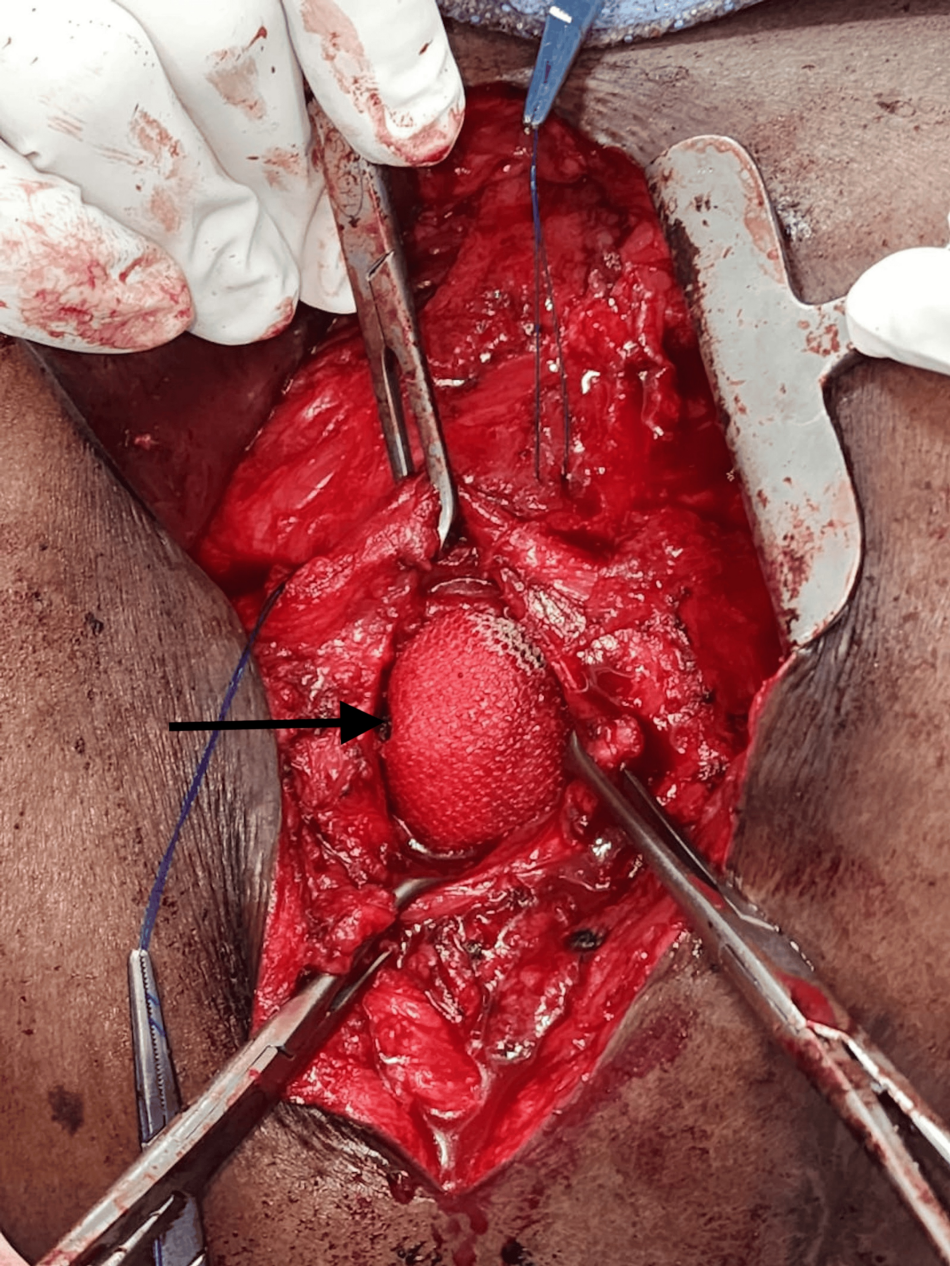
Polypropylene mesh placed extraperitoneally (arrow)

All routine investigations were completed and found to be within the normal range. Investigations showed a leukocyte count of 6.5 × 10^9^/L and a hemoglobin level of 13.8 g/dL. After obtaining anesthesia clearance, the patient underwent onlay mesh repair under general anesthesia. Following sterile aseptic precautions, the patient was placed in the left lateral position, and a horizontal incision was made in the right lumbar region over the swelling. After deepening the incision, all layers were opened, and the hernial sac was identified. A swelling measuring 10 × 8 cm was found bulging through a narrow ring in the inferior triangle (Figure 3). The sac was opened, revealing extraperitoneal fat and omentum as the contents. A 15 × 7.5 cm polypropylene mesh was fixed using polypropylene sutures in the extraperitoneal space after reducing the contents of the sac (Figure 4).

**Figure 5:**
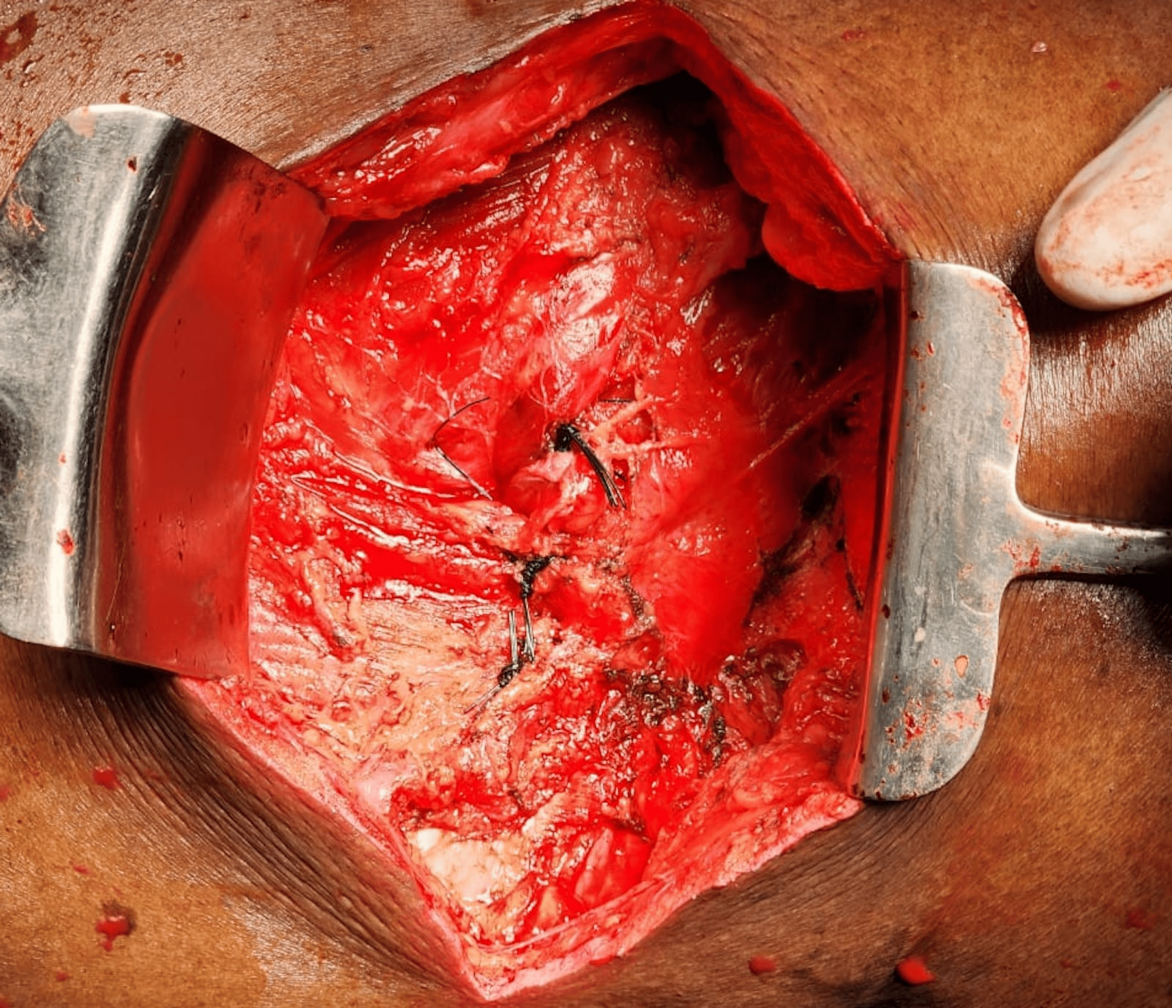
Muscle defect closure using 1-0 Nylon suture material

**Figure 6:**
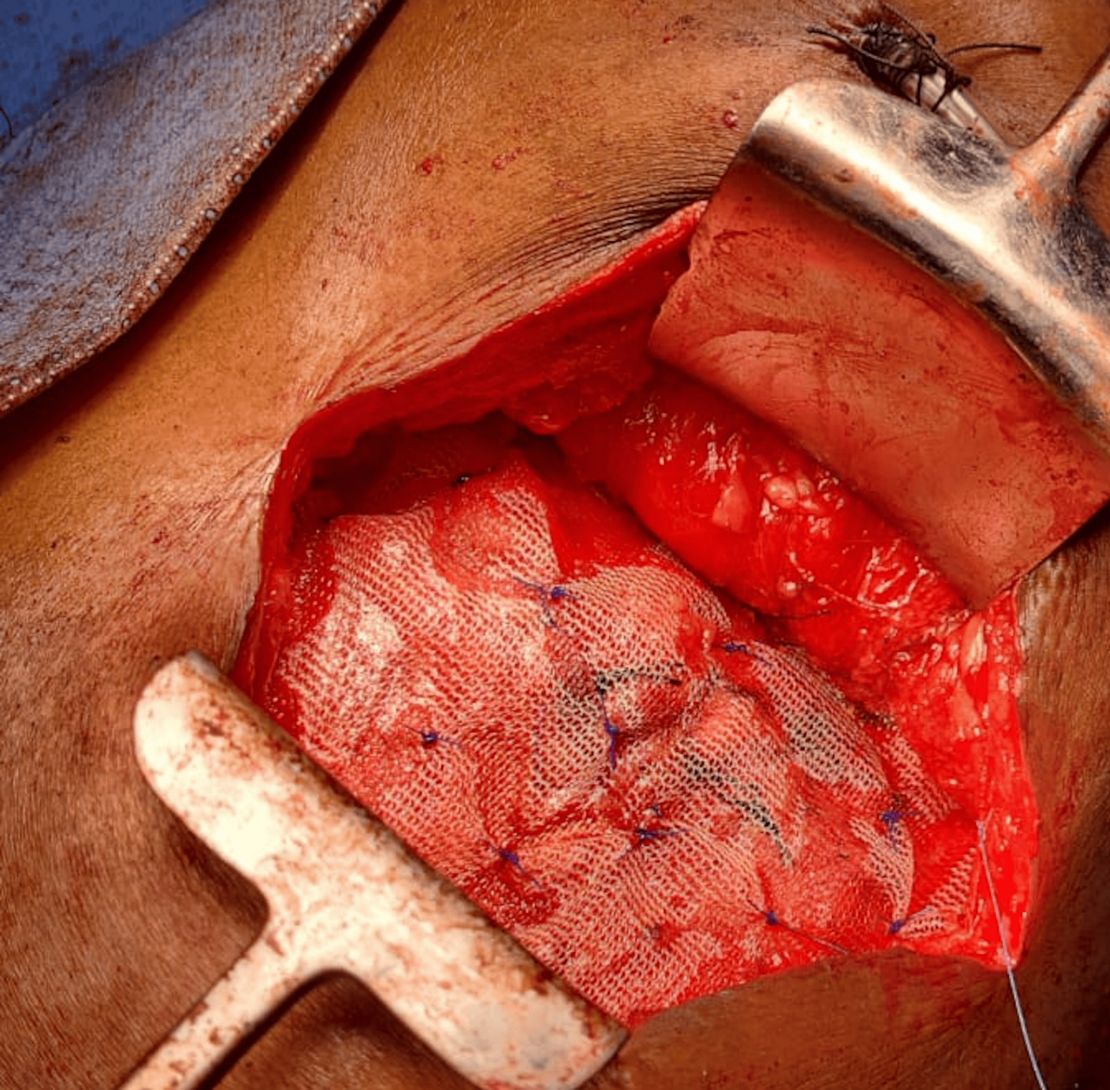
Polypropylene mesh placed over the muscle-sandwich technique

The muscle was repaired using 1-0 nylon interrupted sutures (Figure 5). Over the muscle, a 15 × 15 cm polypropylene mesh was placed and secured with interrupted polypropylene sutures (Figure 6). A negative suction drain was placed, followed by closure of the subcutaneous layer and skin. A sterile compression dressing was applied. Postoperatively, the patient was maintained in the left lateral position until postoperative day 1.

He was advised to mobilize early. Since the postoperative period was uneventful, the drain was removed on day 5, and the patient was discharged. He was followed up regularly for 1.5 years with no signs of recurrence.

## Discussion

The anatomy of the lumbar region includes the lumbar spine, which is composed of five vertebrae, labeled L1 to L5. The posterior part of this region contains muscles such as the erector spinae, quadratus lumborum, and psoas major. The erector spinae muscle extends from the pelvis to the head, aiding in maintaining an upright posture. The quadratus lumborum muscle is located in the lower back and assists in the lateral flexion and extension of the spine. The psoas major muscle, situated deep within the abdominal cavity, plays a role in hip flexion and extension. Together, these muscles and bones form a complex network that supports various functions and movements in the lower back.

Based on etiology, lumbar hernias can be classified into two types: acquired and congenital. Congenital hernias are rare, accounting for only one-fifth of cases, and are often associated with musculoskeletal and rib abnormalities [3]. The majority (8 out of every 10 cases) are acquired, and these can be further divided into primary or secondary [4]. Only around 300 cases of primary lumbar hernias have been reported in the literature, making them a sporadic occurrence [5]. The acquired type typically occurs after procedures such as graft harvesting from the iliac bone, drainage of an iliac abscess, or surgeries in the flank region, including nephrectomy, aortic aneurysm repair, or the use of a latissimus dorsi myocutaneous flap [6].

There are two main types of lumbar hernia. Superior lumbar hernia, also called Grynfeltt hernia, is less common than inferior lumbar hernia, also known as Petit’s hernia. The superior triangle is bordered by the internal oblique muscle in the front, the sacrospinalis muscle posteriorly, the twelfth rib, and the serratus posterior muscle. Petit’s triangle is bounded by the external oblique, latissimus dorsi, and the iliac crest, with the internal oblique forming the floor. The contents of the hernia may include abdominal organs, omentum, retroperitoneal fat, and other structures such as small/large bowel and, in some cases, even the kidneys.

In cases in which palpable swelling is present, ultrasound can aid in confirming the diagnosis [7]. However, a CT scan is considered the preferred diagnostic method [8]. Additionally, reviewing the patient’s medical history may reveal underlying conditions contributing to the development of the hernia. An accurate diagnosis is essential for effective treatment planning and management. Several treatment options are available for lumbar hernias, although nonsurgical interventions are generally reserved for individuals who are not ideal candidates for surgery. Surgical options include primary closure or repair with mesh placement, performed through laparoscopic transabdominal or retroperitoneoscopic methods [9]. The efficacy and outcomes of open and laparoscopic repairs are similar [10]. When planning surgery, it is important to consider the difficulty of clearly defining the margins of the fascial defect and the inherent weakness of the affected structures.

Our patient underwent open mesh repair using the “sandwich technique,” in which two polypropylene meshes were placed - one extraperitoneal and another over the muscle - for additional tensile strength and support. Despite the low recurrence rates associated with this technique, there are only a few case reports highlighting its use [5]. A case series by Sahoo and Anil Kumar, involving four patients with a two-year follow-up, showed that this technique is safe and feasible with no reported recurrences [11].

The patient was advised to limit strenuous activity during the initial recovery period and underwent physical therapy to resume normal activities. The complications of lumbar hernia surgery can vary in severity and may include both intraoperative and postoperative issues. Intraoperative complications include potential damage to structures such as the large intestine and ureter. Postoperative complications can range from wound infection to deep vein thrombosis. Recurrence of the hernia is possible, particularly in patients of advanced age, those with comorbidities, or in cases in which a faulty technique is employed.

A review of the literature published by Köckerling et al. on the sandwich technique for repairing large incisional hernias showed a high complication rate of more than 65% largely comprising of wound infection, superficial skin edge necrosis, and seroma. However, our case showed no complications up to 18 months post-surgery [12].

## Conclusions

Lumbar hernias in the inferior triangle are a rare but potentially dangerous type of hernia that present challenges in both diagnosis and treatment. Advances in medical imaging technology have facilitated more prompt and accurate diagnoses. The sandwich technique for lumbar hernia repair is simple, safe, and practical. In our case, this resulted in no postoperative complications, and the patient was able to resume routine activities. However, further research and studies are necessary to evaluate its long-term effectiveness and outcomes.
